# Harnessing spin precession with dissipation

**DOI:** 10.1038/ncomms10451

**Published:** 2016-01-27

**Authors:** A. D. Crisan, S. Datta, J. J. Viennot, M. R. Delbecq, A. Cottet, T. Kontos

**Affiliations:** 1 Laboratoire Pierre Aigrain, Ecole Normale Supérieure-PSL Research University, CNRS, Université Pierre et Marie Curie-Sorbonne Universités, Université Paris Diderot-Sorbonne Paris Cité, 24 rue Lhomond, 75231 Paris, France

## Abstract

Non-collinear spin transport is at the heart of spin or magnetization control in spintronics devices. The use of nanoscale conductors exhibiting quantum effects in transport could provide new paths for that purpose. Here we study non-collinear spin transport in a quantum dot. We use a device made out of a single-wall carbon nanotube connected to orthogonal ferromagnetic electrodes. In the spin transport signals, we observe signatures of out of equilibrium spin precession that are electrically tunable through dissipation. This could provide a new path to harness spin precession in nanoscale conductors.

## Abstract (web summary)

Control over the orientation of electronic spins forms the basis for spintronic devices in both classical and quantum systems. Here, the authors observe electrically-tunable dissipation-controlled spin precession in a carbon nanotube quantum dot bridging two non-collinearly magnetized electrodes.

## Graphical abstract

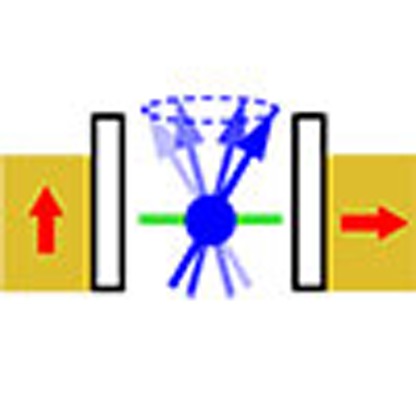

Spin transport is deeply modified if one shrinks spintronics devices down to the nanoscale. This could allow one to envision spin-based devices with new functionalities, such as spin-field effect transistors^1^,^2^,^3^. For devices exploiting actively the electronic spin, however, control over classical or quantum spin rotations has to be achieved. In standard spintronics devices, one usually strives to control the angle of the spin rotations by controlling their speed. Is it possible to alternatively tune the dissipation viewed by the spins to harness their precession angle?

Manipulating spins or magnetizations requires the application of a torque. This can be conveniently done via real- or effective magnetic fields. In the past decade, it has been found that spin-polarized current could also be used, thanks to the spin-transfer torque phenomenon^4^. So far, the spin-transfer torque has been studied in nanopillars^5^, metallic quantum point contacts^6^ or magnetic wires^7^ that is, in conductors enclosing many conduction channels. In these cases, spin transport is classical as the motion of charges is essentially diffusive. Similarly to the spin independent case, one expects qualitative changes if these classical conductors can be replaced by quantum conductors such as nanowires or quantum dots, which display size quantization effects (discrete orbital levels) or charge quantization effects (Coulomb blockade). This would give new paths for spin manipulation in nanoscale systems^8^,^9^,^10^,^11^,^12^,^13^,^14^,^15^,^16^,^17^,^18^.

Here we implement a single-quantum dot connected to leads with non-collinear magnetizations. The device acts like a spin-valve with a tunneling magnetoresistance. While the linear-spin-dependent transport displays the usual signatures of electronic confinement^1^,^2^,^19^, the finite bias magnetoresistance displays a striking antisymmetric reversal with the dot bias voltage. This phenomenon is accompanied by a linear dispersion of the zero magnetoresistance point in the bias-field plane. The slope of the dispersion corresponds to a gate tunable field-to-energy conversion constant ranging from 200 to 700. Such high values cannot be understood in terms of Zeeman or magnetic-field-induced orbital effects. They point to a dissipative control of spin precession. We can account quantitatively for our findings using a simple spin-precession theory and a bias-dependent spin decoherence. These findings illustrate that although dissipation is usually considered as a nuisance, it can become a resource if it is electrically tunable. This could be used as a resource for spin-flip transistors^20^ and could contribute to the understanding of spin-polarized tunneling in magnetic adatoms, molecules or nanoparticles.

## Results

### Device and spin signal in the linear regime

**Figure 1:**
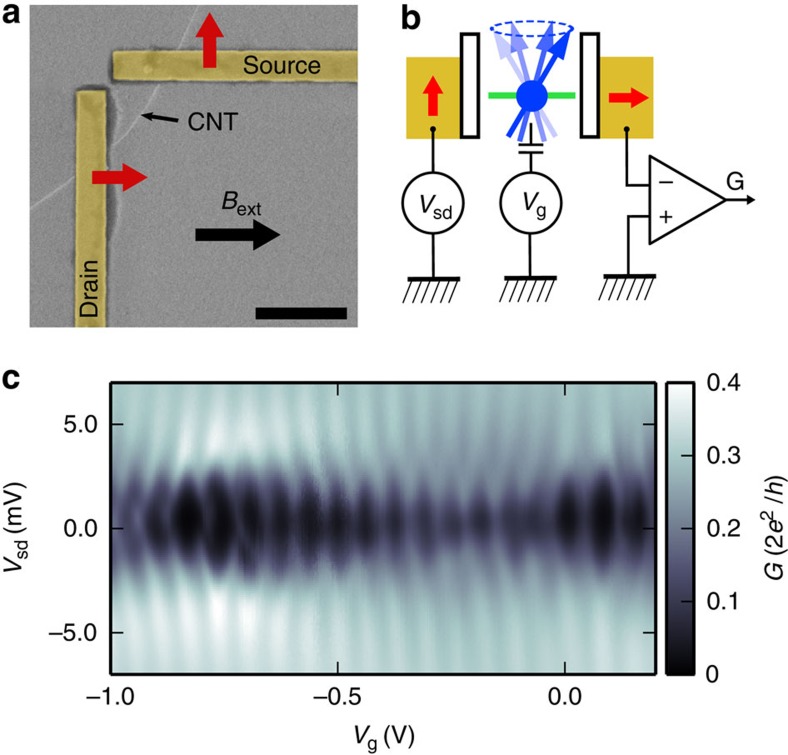
The set-up and its spectroscopy. (**a**) False colours SEM micrograph of a typical device. Two PdNi strips with transverse magnetic anisotropy form a *π*/2 angle. They contact electrically a single-wall carbon nanotube. The red arrows indicate the direction of the magnetizations. A back-gate electrode (not visible) is used to tune the energy levels of the device. As shown by the black thick arrow, the external *B*-field is applied along one of the easy axis of the two PdNi strips. Scale bar, 1 μm. (**b**) Schematics of the principle of the device. The dot carries a spin that can take in principle any direction due to the non-collinear electrodes. The system is probed by the d.c. spin-dependent current flowing through the device. (**c**) Transport spectroscopy of the device presented in the paper showing Coulomb blockade features.

We use carbon nanotubes to fabricate quantum dots with ferromagnetic electrodes^1^,^2^,^3^,^21^,^22^,^23^. Owing to the transverse anisotropy of our ferromagnetic electrodes^24^, one can obtain quantum dots with non-collinear leads by defining with e-beam lithography magnetic electrodes forming an angle, as shown in Fig. 1a, where we display a SEM image of a typical sample in false colours (see Methods). As shown in Fig. 1a, the two electrodes form an angle *π*/2. This results *a priori* to spin precession, as sketched in Fig. 1b. We present here a full transport study of one device, which was stable enough to conduct systematic spin transport measurements at finite bias voltage.

Figure 1c displays the colour scale differential conductance map of the device as a function of the source-drain bias *V*_sd_ and the gate voltage *V*_g_. The colour scale plot displays the characteristic diamonds of the spectroscopy of a quantum dot in the weak Coulomb blockade regime. From the mean height of the diamonds, one can read off a charging energy of about 3 meV. Besides, the variations in the size of the Coulomb diamonds reveals a finite intrinsic level spacing between the dot levels, of the order of 1 meV. Throughout the paper, the temperature is *T*=1.8 K.

**Figure 2:**
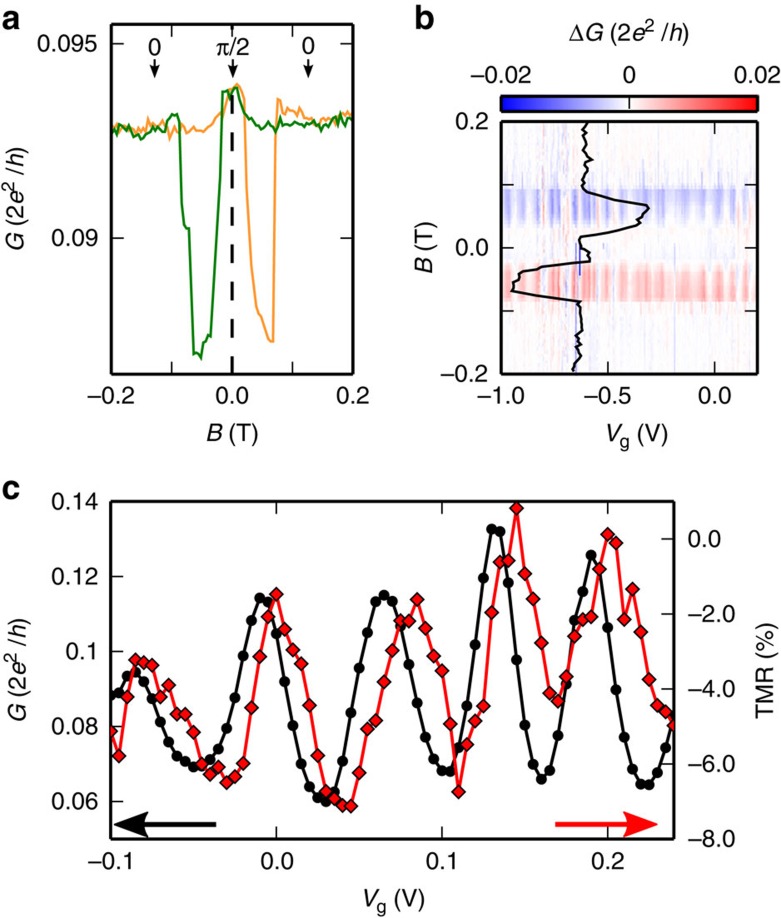
Gate response of magnetoresistance. (**a**) Single-magnetoresistance curve for *V*_sd_=0 mV and *V*_g_=−1.2 V. The orange curve corresponds to increasing magnetic field (*G*_trace_). The green curve corresponds to decreasing magnetic fields (*G*_retrace_). (**b**) Wide gate-voltage response of the spin signal Δ*G*=*G*_trace_−*G*_retrace_ in colour scale as a function of the *B*-field and the gate voltage *V*_g_. The black line corresponds to a cut at *V*_g_=−0.85 *V* (**c**) Left axis, black circles: linear conductance modulations at zero magnetic field as a function of the back-gate voltage. Right axis, red diamonds: TMR modulations measured at 75 mT simultaneously with the linear conductance.

We first study spin transport in the linear regime (*eV*_sd_<*k*_B_*T*), *e* being the elementary charge and *k*_B_ the Boltzman constant. Figure 2a displays the typical magnetoresistance signal observed when we apply an external magnetic field along one of the electrodes and perpendicular to the other one, as sketched in Fig. 1a. We observe a spin-valve behaviour with hysteretic switchings at about 10 and 80 mT on increasing the magnetic field and − 10 and −80 mT on decreasing the magnetic field. The angle *φ* between the two magnetizations is *π*/2 at zero magnetic field, as characterized by room temperature magnetic force microscopy (not shown) and 0 at high magnetic field. In the geometry we are using, the first switching event can be attributed to a switch from *φ*=*π*/2 to ≈*π* due to the progressive rotation of the left (L) electrode, and the second sharper switching event can be attributed to a switch from ≈*π* towards 0 due to a switch of the right(R) electrode. The spin-dependent signal may be defined in the conventional way, using the *TMR*=(*G*_retrace_−*G*_trace_)/*G*_retrace_ at a magnetic field *B*=75 mT. Here *G*_trace_ is the conductance on increasing the external magnetic field and *G*_retrace_ is the conductance on decreasing the external magnetic field. Note that we do not know the exact magnetic configuration for *B*=75 mT. However, it is *a priori* non-collinear due to the progressive rotation of the magnetization orthogonal to the field orientation at zero field. This implies that *G*_retrace_ does not correspond to an angle of 0 at *B*=75 mT but to an angle between 0 and *π*/2. This can be supported by numerical simulations of the magnetization reversal (not shown). For simplicity we will use *π*/2 in our interpretation, knowing that a smaller angle would modify only quantitatively the interpretation and give a more optimistic evaluation for the spin-relaxation rate, which we will estimate below. One may also define Δ*G*=*G*_trace_−*G*_retrace_, which is more direct than the *TMR* in the out of equilibrium regime for non-linear devices such as quantum dots. In Fig. 2b, the amplitude of Δ*G* is represented in colorscale plot as a function of *B* and *V*_g_. The clear vertical red (positive) and blue (negative) stripes show that the hysteresis is modulated regularly as the gate voltage is swept. This behaviour is similar to the one observed in the collinear regime^1^,^2^. The *TMR* is represented in Fig. 2c together with the linear conductance at zero field. Both quantities oscillate as a function of the gate voltage due to quantum interference as well as interactions in the device^25^. The oscillations of the *TMR* are slightly phase shifted (by about *π*/4) from those of the conductance, with the same period. This is typical of spin injection in a coherent conductor^26^ with quantized energy levels. It allows us to discard spurious mechanisms leading to a hysteresis, like the magnetocoulomb effect, which would imply that the modulations of the TMR would be the derivative of those of the conductance^19^,^27^.

### Spin signal in the non-linear regime

**Figure 3:**
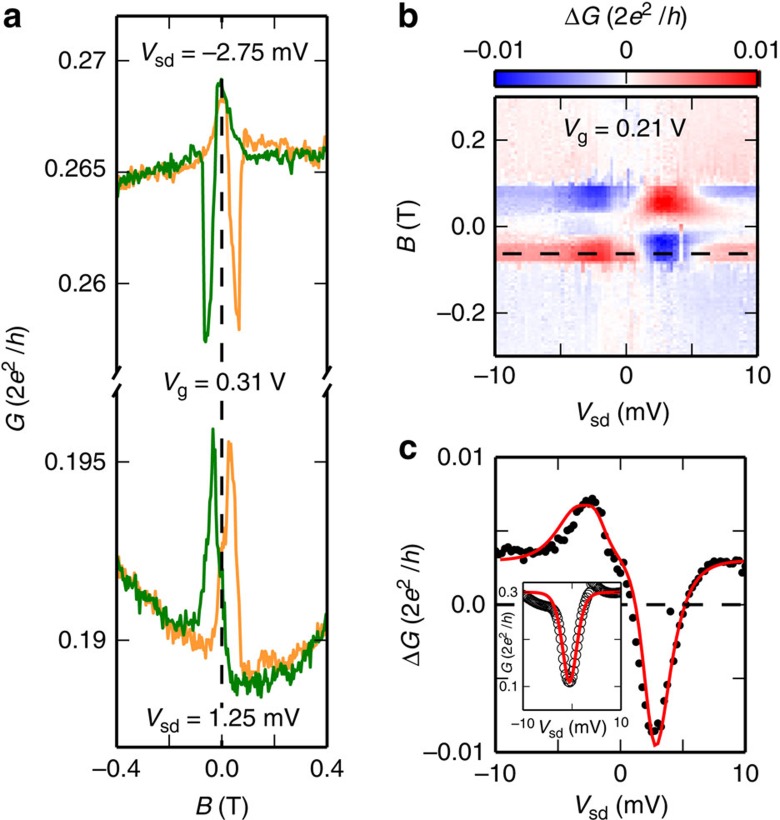
Bias-dependent spin signal. (**a**) Single-magnetoresistance curves for *V*_sd_=−2.75 mV and *V*_sd_=1.25 mV. The back-gate voltage is *V*_g_=0.31 V. (**b**) Colour scale plot of the spin signal Δ*G* as a function the *B*-field and the source-drain bias *V*_sd_ for *V*_g_=0.21 V. (**c**) Measured Δ*G* as a function of the source-drain bias *V*_sd_ for *V*_g_=0.21 V and *B*=−75 mT (black circles), and prediction from equations (1)–(3), 2, 5 (red solid line). Inset: differential conductance in open circles, interpolated by the red line according to the function given in the main text.

We now discuss spin transport in the non-linear regime (*eV*_sd_>*k*_B_*T*). The main results are shown in Fig. 3 where we display the hysteresis and Δ*G* as a function of both *B* and the source-drain bias *V*_sd_ for a constant *V*_g_. The hysteresis changes sign as the bias is changed from positive to negative values. As we show in Fig. 3a, one can revert the sign of the spin signal upon changing the bias from *V*_sd_=−2.75 mV to *V*_sd_=1.25 mV, at *V*_g_=0.31 V. The magnetoresistance reversal is best seen in Fig. 3b in colour scale of Δ*G* as a function of the *B* field and *V*_sd_ for a constant *V*_g_. For positive values of *B*, we see a blue stripe for *V*_sd_<0 and a dominantly red stripe for *V*_sd_>0, showing that the spin signal has approximately the same symmetry as the current. Such a behaviour is shown in details in Fig. 3c, where Δ*G* versus the bias *V*_sd_ is represented for constant values of *V*_g_ and *B*. The spin signal Δ*G* displays a nearly antisymmetric behaviour with *V*_sd_. This suggests out of equilibrium spin precession, as we discuss below. On very general arguments relying on the high asymmetry between the L and R contacts(see Methods), the spin signal Δ*G* can be very simply written as an expansion of the projection of the dot's spin on the magnetization of the more opaque tunnel barrier. Defining the opaque barrier as left(L) barrier, we obtain:


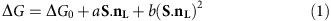


The above equation implies that the angle dependence of the conductance contains both cos *φ* and (cos *φ*)^2^ terms with all possible relative signs. This is already crucial to understand our magnetoresistance traces such as those of Figs 2a and 3a. In particular, the (cos *φ*)^2^ term allows us to understand how *G*(*π*/2) can be in between or larger than *G*(0) and *G*(*φ*≈*π*). In this framework, our measurements can be understood using a simple semiclassical Bloch–Redfield equation for the spin on the dot, which allows one to calculate *S*_*L*_=**S**.**n**_**L**_ (see Methods). We obtain:


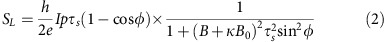


**Figure 4:**
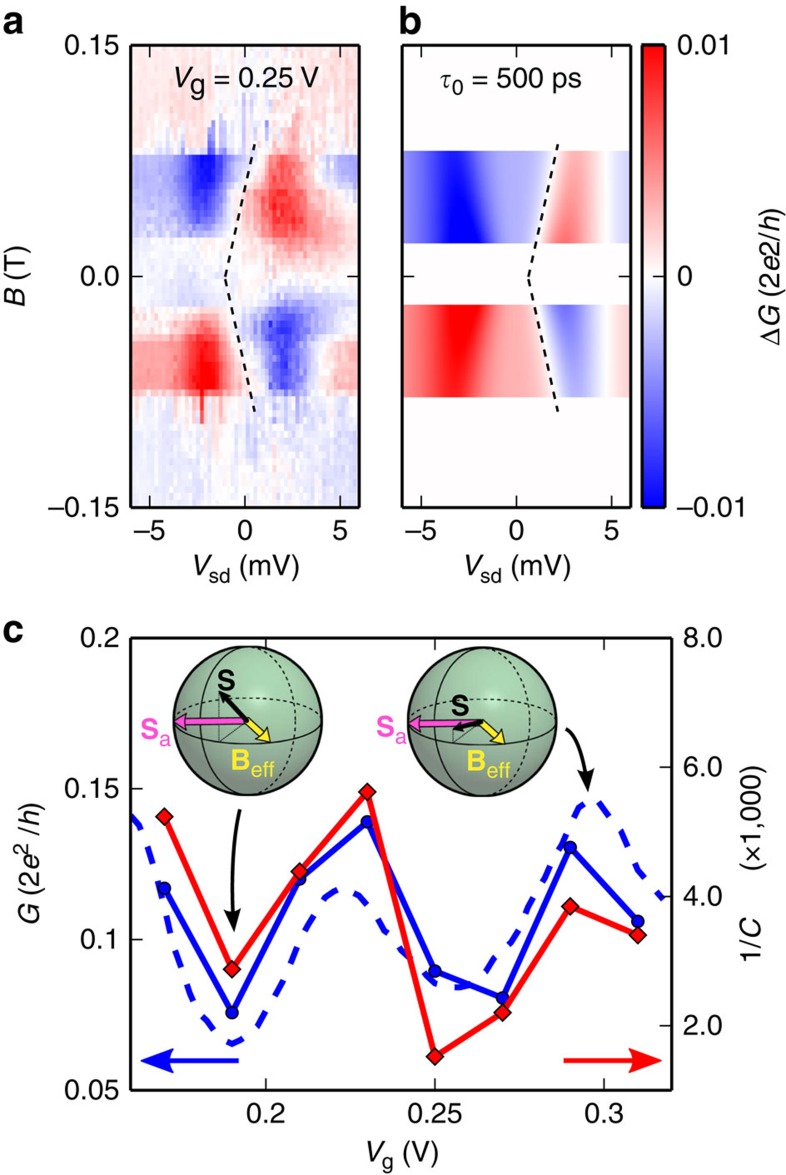
Bias-controlled spin precession. (**a**) Zoom on the colour scale plot of the spin signal as a function the *B*-field and the source-drain bias *V*_sd_ for *V*_g_=0.25 V. The tilted white line highlighted by the black dashed line corresponds to the points where the spin signal changes sign. (**b**) Simulated colour scale plot of the spin signal as a function the *B*-field and the source-drain bias *V*_sd_ for *V*_g_=0.25 V using equations (1)–(3), 2, 5, *τ*_0_=500 ps and *V*_0_=2.2 mV. (**c**) Red diamonds: inverse field to energy conversion constant 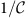 (right axis) as a function of gate voltage. Blue circles and blue dashed line: Linear conductance measured simultaneously with the spin signal (circles) and taken from a horizontal cut of the colour scale plot of Fig. 1c (dashed line). The latter has been shifted by −0.02 V to compensate the small gate shift, which occurred between the two measurements. Inset: schematics of the competition between spin accumulation, spin precession and spin relaxation.

Here *I* is the electrical current flowing through the device, *p* is the spin polarization of current, *κ*=+1 for *B*>0 and *κ*=−1 for *B*<0, *κB*_0_ is the effective magnetic field arising from the right (R) magnetization which follows the external magnetic field *B* and *τ*_s_ is the total relaxation time of the dot's spin. Considering the switching sequence in the experiment, it is suitable to assume *κ*=±1 to interpret the TMR measurements. Throughout the paper, we use *φ*=*π*/2 in our modelling. Equation (2) has a simple interpretation: the first part stems from the competition between spin accumulation and spin relaxation and the second part (which has a Lorentzian shape as a function of *B*) is a Hanle type term, which describes spin precession. As sketched in Fig. 4b in the inset, it is *B*_eff_=*B*+*κB*_0_ and *τ*_s_, which controls this spin precession. The effective field tends to pull out of plane the spin ***S*** towards the north pole of the Bloch sphere, whereas the spin relaxation tends to push it in plane. We can therefore straightforwardly compare Equation (2) with our data provided we know *τ*_s_. Importantly, we work with carbon nanotubes where a low intrinsic spin relaxation rate is expected (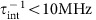)^28^. However, we use tunnel contacts which are not very opaque (tunnel rates >100 GHz). Hence, the contacts can also contribute to spin relaxation (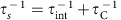), in principle. In a diffusive metallic dot, it has been shown theoretically that a ferromagnetic contact can induce an effective field inside the dot, but also an effective spin relaxation^29^,^30^. The parameters *B*_0_ and *τ*_C_ correspond to the generalization of these concepts to quantum dots with a finite intrinsic level spacing^25^,^26^,^31^,^32^,^33^,^34^. Note that *B*_0_ has already been observed experimentally in quantum dots with ferromagnetic contacts^1^,^3^, but the contact-induced spin relaxation time *τ*_C_ has raised little attention so far for quantum dots. One naturally expects *τ*_C_ to be drastically different whether the dot's level is at resonance or off resonance due to the quantum confinement of electrons and Coulomb blockade, which leads a strong energy dependence of the dot density of states^32^,^33^,^34^. This behaviour implies that we can control the spin-precession angle by simply changing the bias voltage.

In order to characterize *τ*_s_, we interpret quantitatively the data of Fig. 3c. More precisely, we use Equations (1) and (2) with *a*=−0.012, *b*=−0.0096,


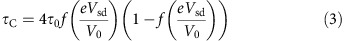


*f*(*E*) the Fermi-Dirac function, 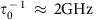, *V*_0_=2.2 meV, and 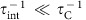. This gives the solid red curve fitting quantitatively the Δ*G*(*V*_sd_) curve for *V*_g_=0.21 V in Fig. 3c. The high value of 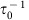 confirms that *τ*_s_ is indeed dominated by *τ*_C_. Besides, the bias dependence of *τ*_s_ matches with the interpolation of the bias dependence of *G* shown in the inset of Fig. 3b, i.e. 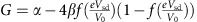. This again points to a contact-induced spin relaxation effect.

### Out of equilibrium spin precession

Spin accumulation tends to force Δ*G* to follow the behaviour of the current (and therefore to be antisymmetric in *V*_sd_), whereas spin relaxation acts against it. The balance between these two effects sets the high-voltage limit of Δ*G*, visible in Fig. 3c. In our case, from Equations (1), (2) and (3), at high voltages, Δ*G* is symmetric that is, Δ*G*(±*V*_sd_)=Δ*G*_0_ since *Iτ*_s_ and thus **S** vanish. Spin precession can affect Δ*G* only in the intermediate voltage bias regime, for which **S** is finite. Hence, we now make a closer inspection of the intermediate voltage bias regime to show that out of equilibrium spin precession is indeed present in our experiment. Figure 4a displays a similar colorscale plot as in Fig. 3b but zoomed between −6 and 6 mV and for *V*_g_=0.25 V. As highlighted by the tilted green dashed lines, we observe a dispersion of the zero Δ*G* point in the *B*−*V*_sd_ plane. This slope is very well reproduced by Equations (2) and (3) as one can see in Fig. 4b. We account for the switching of the magnetizations with simple Heaviside functions, which lead to the horizontal stripes. The slope of the white line denoting the vanishing of the Δ*G* stems from the second (precession) part of equation (2) and allows us to extract 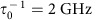, and *B*_0_=20 mT. As the bias is increased the spin relaxation time decreases leading to a larger width of the Lorentzian part of equation (2). This confirms the ansatz (3). For testing it further, it is useful to define a field-to-energy conversion constant 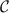, 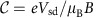, from the slope of the white line for each gate voltage, *μ*_B_ being the Bohr magneton. The obtained values range from 200 to 700. This means that the small field of the order of 100 mT applied on our device has an effect equivalent to 20 to 70 T considering a standard *g*-factor of 2. Hence, Zeeman or orbital effects are too weak to explain this feature. Such high values of 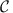 can only be explained if spin precession is taking place in the device. The gate modulations of 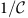 as well as the conductance modulations are shown in Fig. 4c. One can see very strong correlations between *G* and 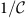, which are very well accounted for by our model using again Equations (1)–(3), 2, 5. This confirms the interpretation of our data in terms of dissipative control of spin precession. This is sketched in Fig. 4c in inset. The spin relaxation is directly linked to the conductance via the density of states and has the same bias-gate dependence as the conductance. When the conductance is high, the spins relax faster and the out-of-plane spin component is small (right Bloch sphere). When the conductance is low, the spins relax slower and the out-of-plane spin component is large (left Bloch sphere). The slope 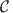 is roughly given by *Bτ*_s_≈const, which is a level line of the Hanle term in equation (2). This qualitatively accounts for the strong correlations observed between 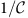 and *G*.

## Discussion

The out of equilibrium precession presented above relies, besides the non-collinear geometry, on a bias and gate-dependent spin relaxation, which directly maps onto the conductance. The use of the spin relaxation for harnessing the overall spin precession angle is appealing for spintronics applications since it does not demand a very closed quantum dot with long-spin relaxation times. On the contrary, an open quantum system can be used where dissipation is engineered to control the spin degree of freedom. This could be used for implementing spin-flip transistor-like spintronic devices or fast initialization of single spins for quantum information.

## Methods

### Experimental

The devices nanofabricated for this study are based on single-wall carbon nanotubes grown by chemical vapour deposition with a methane process on a highly doped Si substrate used as a back gate^23^. The samples are connected to 30-nm thick *Ni*_0.7_*Pd*_0.3_ contacts forming an angle of *π*/2. They are coated with a 5-nm thick *Pd* layer. The angle between the magnetizations of the electrodes is checked on control samples at room temperature with a magnetic force microscope. The conductance measurements are done with the standard lock-in detection technique with an a.c. modulation of 100 μV at 777.77 Hz. Each magnetoresistance plot is obtained by averaging four times single curves (which all display the hysteresis switching).

### Bloch–Redfield-like equations for the spin dynamics of the dot's spin

The spin accumulation on the dot is of the form 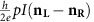 where *p* is the spin polarization of the left and right contacts (taken to be equal here), *I* is the electrical current and **n**_L(R)_ are the unit vectors collinear to the left (L) magnetization and the right (R) magnetization, respectively^33^. We assume that the spin **S** of the dot is subject to a magnetic field (real or effective) **B**=*B*_L_**n**_L_+*B*_R_**n**_R_ in the plane of the magnetizations (our experimental situation). The Bloch–Redfield type equations describing the dynamics of **S** read, in the limit of weakly polarized contacts:


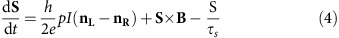


Defining **S**=*S*_*L*_**n**_L_+*S*_*R*_**n**_R_+*S*_⊥_**n**_L_ × **n**_R_ and cos *φ*=**n**_L_.**n**_R_, we get, in the stationary regime:


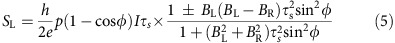


The above formula yields equation (2) of the maintext provided *B*_L_=0, which is reasonable since the L contact is very weakly coupled to the dot and *B*_R_=*B*+*κB*_0_, with *κ*=|*B*|/*B* and *B*_0_ an effective field induced by the right contact.

### Relation between the current through the dot and spin accumulation

The conductance peaks of the device are significantly smaller than *e*^2^/*h,* while their width is much larger than temperature. This indicates that the two tunnel contacts are asymmetric. Hence, we can assume that the left contact has a low transparency. In this regime, the current through the device can be expressed using a simplified Meir–Wingreen formula:


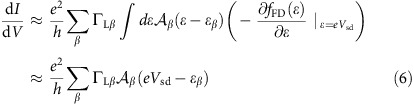


where *β*=± corresponds to the spin quantization axis along the L magnetic electrode coupled to the quantum dot with the coupling strength Γ_L*β*_=Γ_L0_(1+*βP*_L_) and 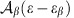 is the density of states per spin direction *β* in the quantum dot. Here *ɛ*_*β*_ is assumed to depend on the gate voltage *V*_g_, on the external magnetic field **B** and on the spin accumulation. We use the standard approach where the spin accumulation **S** produces an effective Zeeman splitting, which directly affects the dot density of states^35^,^36^. We can therefore write: 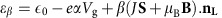 with *α* the gate lever-arm. The constant *J* is a phenomenological electronic spin-macrospin interaction constant. It has the dimension of a pulsation. The constant *μ_B_* is the Bohr magneton. Note that equation (6) implicitly assumes that *ħJ*<<*eV*_0_ (*V*_0_ is defined in the main text) since it neglects the term in the differential conductance arising from the derivative of **S** with respect to bias *V*. This inequality is implied by our data since the observed TMR signals are of the order of few %.

Developing the above expression at second order in (*J***S**+*μ*_B_**B**).**n**_L_, we get:


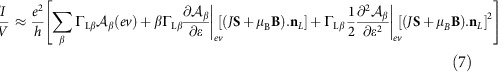


with 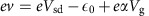. We will assume below that *B*_*L*_=**B**.**n**_L_=0, that is, the magnetic field is perpendicular with the weakly transparent contact (we have checked that the case **B**.**n**_*R*_=0 is not consistent with out data). For *φ*=0, one has **S**=0 (no spin accumulation). Hence, one can interpret our data using (up to a global sign depending on *B*):


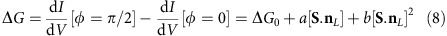


with


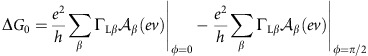



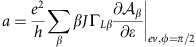



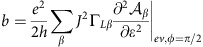


In our approach, the high-voltage limit of Δ*G* is given by Δ*G*_0_ since *Iτ*_s_ and thus **S** vanish at high voltages.

## Additional information

**How to cite this article**: Crisan, A. D. *et al.* Harnessing spin precession with dissipation. *Nat. Commun.* 7:10451 doi: 10.1038/ncomms10451 (2016).

## References

[b1] Sahoo S. *et al.* Electric field control of spin transport. Nat. Phys. 1, 99–102 (2005).

[b2] Man H. T., Wever I. J. W. & Morpurgo A. Spin-dependent quantum interference in single-wall carbon nanotubes with ferromagnetic contacts. Phys. Rev. B 73, 241401 (2006).

[b3] Hauptmann J. R., Paaske J. & Lindelof P. E. Electric field controlled spin reversal in a quantum dot with ferromagnetic contacts. Nat. Phys. 4, 373–376 (2008).

[b4] Brataas A., Bauer G. E. W. & Kelly P. J. Non-collinear magnetoelectronics. Phys. Rep. 427, 157–255 (2006).

[b5] Myers E. B., Ralph D. C., Katine J. A., Louie R. N. & Buhrman R. A. Current-induced switching of domains in magnetic multilayer devices. Science 285, 867–870 (1999). 10436150 10.1126/science.285.5429.867

[b6] Chen T. Y., Ji Y., Chien C. L. & Stiles M. D. Current-driven switching in a single exchange-biased ferromagnetic layer. Phys. Rev. Lett. 93, 026601 (2004). 15323935 10.1103/PhysRevLett.93.026601

[b7] Wegrowe J. E. *et al.* Current-induced magnetization reversal in magnetic nanowires. Europhys. Lett. 45, 626–632 (1999).

[b8] König J. & Martinek J. Interaction-driven spin precession in quantum-dot spin valves. Phys. Rev. Lett. 90, 166602 (2003). 12731989 10.1103/PhysRevLett.90.166602

[b9] Braun, König J. & Martinek J. Theory of transport through quantum-dot spin valves in the weak-coupling regime. Phys. Rev. B 70, 195345 (2004).

[b10] Rudzinski W., Barnas J., Swirkowicz R. & Wilczynski M. Spin effects in electron tunneling through a quantum dot coupled to noncollinearly polarized ferromagnetic leads. Phys. Rev. B 71, 205307 (2005).

[b11] Wetzels W. *et al.* Exchange effects on electron transport through single-electron spin-valve transistors. Phys. Rev. B 74, 224406 (2006).

[b12] Waintal X. & Parcollet O. Current induced spin torque in a nanomagnet. Phys. Rev. Lett. 94, 247206 (2005).

[b13] Koller S., Mayrhofer L. & Grifoni M. Spin transport across carbon nanotube quantum dots. New J. Phys. 9, 348 (2007).

[b14] Barnas J. & Weymann I. Spin effects in single-electron tunnelling. J. Phys. Cond. Matter 20, 423202 (2008).

[b15] Cottet A. Gate-dependent spin-torque in a nanoconductor-based spin-valve. Phys. Rev. B 84, 054402 (2011).

[b16] Lim J.-S., López R., Giorgi G.-L. & Sánchez D. Kramers polarization in strongly correlated carbon nanotube quantum dots. Phys. Rev. B 83, 155325 (2011).

[b17] Gaas M. *et al.* Universality of the Kondo effect in quantum dots with ferromagnetic leads. Phys. Rev. Lett. 107, 176808 (2011). 22107560 10.1103/PhysRevLett.107.176808

[b18] Hoffman S. & Tserkovnyak Y. Magnetic exchange and nonequilibrium spin current through interacting quantum dots. Phys. Rev. B. 91, 245427 (2015).

[b19] Cottet A. *et al.* Nanospintronics with carbon nanotubes. Semicond. Sci. and Technol. 21, S78–S95 (2006).

[b20] Urban D., Braun M. & König J. Theory of a magnetically-controlled quantum-dot spin transistor. Phys. Rev. B 76, 125306 (2007).

[b21] Tombros N., van der Molen S. J. & van Wees B. J. Separating spin and charge transport in single-wall carbon nanotubes. Phys. Rev. B 73, 233403 (2006).

[b22] Hueso L. E. *et al.* Transformation of spin information into large electrical signals using carbon nanotubes. Nature 445, 410–413 (2007). 17251975 10.1038/nature05507

[b23] Feuillet-Palma C. *et al.* Conserved spin and orbital phase along carbon nanotubes connected with multiple ferromagnetic contacts. Phys. Rev. B 81, 115414 (2010).

[b24] Chauleau J. Y. *et al.* On the magnetization textures in NiPd nanostructures. Phys. Rev. B 84, 094416 (2011).

[b25] Cottet A. & Choi M.-S. Magnetoresistance of a quantum dot with spin-active interfaces. Phys. Rev. B 74, 235316 (2006).

[b26] Cottet A. *et al.* Controlling spin in an electronic interferometer with spin-active interfaces. Europhys. Lett. 74, 320–326 (2006).

[b27] Datta S. *et al.* Magneto-Coulomb effect in carbon nanotube quantum dots filled with magnetic nanoparticles. Phys. Rev. Lett. 107, 186804 (2011). 22107663 10.1103/PhysRevLett.107.186804

[b28] Viennot J. J., Dartiailh M. C., Cottet A. & Kontos T. Coherent coupling of a single spin to microwave cavity photons. Science 349, 408–411 (2015). 26206930 10.1126/science.aaa3786

[b29] Cottet A., Huertas-Hernando D., Belzig W. & Nazarov Y. V. Spin-dependent boundary conditions for isotropic superconducting Green's functions. Phys. Rev. B 80, 184511 (2009).

[b30] Zaffalon M. & van Wees B. J. Spin injection, accumulation, and precession in a mesoscopic nonmagnetic metal island. Phys. Rev. B 71, 125401 (2005).

[b31] Martinek J. *et al.* Kondo effect in quantum dots coupled to ferromagnetic leads. Phys. Rev. Lett. 91, 127203 (2003). 14525397 10.1103/PhysRevLett.91.127203

[b32] Cottet A., Belzig W. & Bruder C. Positive cross-correlations due to dynamical channel blockade in a three-terminal quantum dot. Phys. Rev. B 70, 115315 (2004). 10.1103/PhysRevLett.92.206801 15169373

[b33] Braun M., König J. & Martinek J. Hanle effect in transport through quantum dots coupled to ferromagnetic leads. Europhys. Lett. 72, 294–300 (2005).

[b34] Schulenborg J., Splettstoesser J., Governale M. & Contreras-Pulido L. D. Detection of the relaxation rates of an interacting quantum dot by a capacitively coupled sensor dot. Phys. Rev. B 89, 195305 (2014).

[b35] van Son P. C., van Kempen H. & Wyder P. Boundary resistance of the ferromagnetic-nonferromagnetic metal interface. Phys. Rev. Lett. 58, 2271–2273 (1987). 10034698 10.1103/PhysRevLett.58.2271

[b36] Valet T. & Fert A. Theory of the perpendicular magnetoresistance in magnetic multilayers. Phys. Rev. B 48, 7099–7113 (1993). 10.1103/physrevb.48.7099 10006879

